# Pharmacogenomics of acetaminophen in pediatric populations: a moving target

**DOI:** 10.3389/fgene.2014.00314

**Published:** 2014-10-14

**Authors:** Anne E. Krasniak, Gregory T. Knipp, Craig K. Svensson, Wanqing Liu

**Affiliations:** ^1^Department of Medicinal Chemistry and Molecular Pharmacology, College of Pharmacy, Purdue UniversityWest Lafayette, IN, USA; ^2^Industrial and Physical Pharmacy, College of Pharmacy, Purdue UniversityWest Lafayette, IN, USA

**Keywords:** acetaminophen, ontogeny, pharmacogenetics, polymorphism, pediatric

## Abstract

Acetaminophen (APAP) is widely used as an over-the-counter fever reducer and pain reliever. However, the current therapeutic use of APAP is not optimal. The inter-patient variability in both efficacy and toxicity limits the use of this drug. This is particularly an issue in pediatric populations, where tools for predicting drug efficacy and developmental toxicity are not well established. Variability in toxicity between age groups may be accounted for by differences in metabolism, transport, and the genetics behind those differences. While pharmacogenomics has been revolutionizing the paradigm of pharmacotherapy for many drugs, its application in pediatric populations faces significant challenges given the dynamic ontogenic changes in cellular and systems physiology. In this review we focused on the ontogenesis of the regulatory pathways involved in the disposition of APAP and on the variability between pediatric, adolescent, and adult patients. We also summarize important polymorphisms of the pharmacogenes associated with APAP metabolism. Pharmacogenetic studies in pediatric APAP treatment are also reviewed. We conclude that while a consensus in pharmacogenetic management of APAP in pediatric populations has not been achieved, a systems biology based strategy for comprehensively understanding the ontogenic regulatory pathway as well as the interaction between age and genetic variations are particularly necessary in order to address this question.

## INTRODUCTION

It has been over half a century since acetaminophen (APAP), or paracetamol, was approved by the United States Food and Drug Administration (FDA) to be used as an analgesic and antipyretic. APAP is commonly used to mitigate mild pain or headache symptoms and is available over the counter in the United States. It is also frequently used in combination with more powerful pain relievers such as hydrocodone or oxycodone via prescription drug products. Despite its wide use, optimal dosing to achieve efficacy and minimize toxicity remains problematic. Thus, the unregulated access and non-compliant use of APAP leading to an overdose is one of the leading causes of drug induced liver injury (Food and Drug Administration, 2014). Since such liver injury is generally progressive, sometimes leading to the need for liver transplantation, the optimal goal is prevention of overdose and subsequent toxicity by increasing awareness. Based on the prevalence of hepatic injury, the FDA recently lowered the maximum daily dose of APAP from 4 g/day to 3 g/day, and has also stated that doses over 325 mg may cause liver toxicity (Food and Drug Administration, 2014). In addition to the dosing change, it is of high interest to identify patients that are most and least susceptible to liver damage from APAP for prophylactic purposes.

APAP is a commonly used fever reducer in pediatric populations. Although numerous dosage forms have been developed for the pediatric population, the current dosage recommendations for APAP in children still largely rely on body weight. However, pediatric patients are not simply small adults. They often require vastly different treatment because of their dynamic physiology that affects the pharmacokinetics (PK), including absorption, distribution, metabolism, and excretion (ADME) of many drugs. Differences in APAP PK, pharmacodynamics (PD), toxicity profile and efficacy between individuals and populations have been widely noted (Ward et al., 2001; Pineiro-Carrerro and Pineiro, 2004; Zuppa et al., 2011; Leonis et al., 2013). While pharmacogenetic and pharmacogenomic approaches have been applied to understand the inter-patient difference in pharmacological phenotypes of many drugs, studies in these areas addressing the variability of APAP PK/PD are still limited. While the complex PK process of APAP might be a significant hurdle for this, it is particularly difficult to apply these approaches in pediatric populations due to the dynamic physiology, sample availability, difficulties in monitoring phenotypes, etc. The ontogenesis of drug ADME pathways plays an important role in determining a patient’s response to the drug. Thus, in deploying APAP as a therapeutic option, one must account for both age-dependent differences in the PK/PD of APAP and the inter-patient variability within the pediatric population itself. Both sources of variability will contribute to the individual susceptibility to toxicity from this widely used agent.

Here we review the major clinical outcome variability for APAP observed between pediatric and adult patients, as well as among children of different ages. We also summarize the progress in understanding the role of genetic alleles and age-related gene expression as it pertains to the phenotypic variability between patients. Lastly, we also highlight the challenges as well as new opportunities in APAP pharmacogenomic research.

## INTER-PATIENT VARIABILITY IN PEDIATRIC APAP PHARMACOLOGY

### TOXICITY AND EFFICACY

Acetaminophen is generally well tolerated, making it an attractive choice for the treatment of fever and pain in the pediatric population. Hepatotoxicity is the greatest concern associated with APAP treatment. A 1981 study showed that younger patients had less hepatotoxicity when exposed to the same levels of APAP acutely as older children and adults (Rumack et al., 1981). However, the pediatric acute liver failure study group has found that in chronic exposure to APAP (in contrast to a single toxic dose), younger patients (median age, 3.5 years) experienced a greater level of hepatotoxicity comparative to older adolescent patients (median age 15.2 years) who were more likely to experience hepatotoxicity from a single toxic dose (Rumack et al., 1981).

**FIGURE 1:**
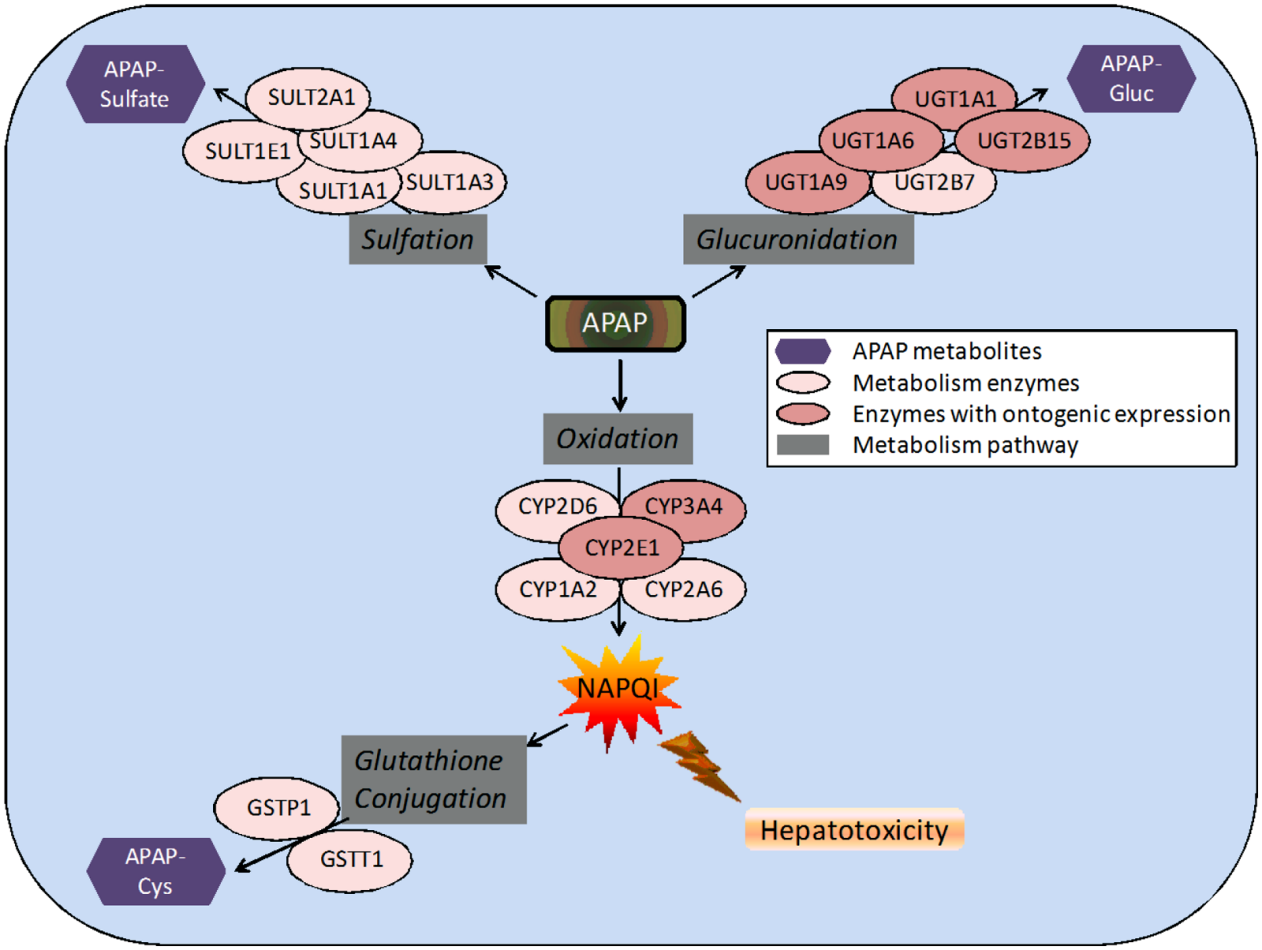
**Pathways and enzymes involved in the metabolism of acetaminophen (APAP).** Note: APAP-Gluc, APAP-glucuronide; APAP-Cys, 3-cysteinyl-APAP.

The reason underlying this age difference in toxicity remains incompletely understood. It has been noted that younger children may be less susceptible to APAP-induced hepatotoxicity because of increased glutathione stores or metabolism differences as a result of genetic variability (Pineiro-Carrerro and Pineiro, 2004). Glutathione synthetase deficiency leads to elevated *N*-acetyl-*p*-benzoquinone imine (NAPQI) and increased hepatotoxicity (Dahlin and Nelson, 1982) (See **Figure 1** for the pathway). The toxicity of APAP is linked closely to its metabolism in the body. A ceiling dose of APAP for children has not been established because therapeutic doses that have been administered to children have caused toxicity in some cases. This may be because conditions vary between children, particularly pharmacogenetic differences, drug interactions, current medical disorders, or malnutrition. Nutritional deficiencies and drug-drug interactions are most likely to cause differences in metabolism and toxicity at normal doses (Ward et al., 2001). Ethnicity also plays a large role in the frequency of functional polymorphisms in pharmacogenes, and the appearance of these pharmacogenes can vary greatly between different ethnic populations (Suarez-Kurtz et al., 2014). These polymorphisms may also account for variability in APAP metabolism.

Besides hepatotoxicity, other APAP side effects have also been observed in children. A cross-sectional study of children 2–6 years of age concluded that frequent APAP use in these patients was associated with the formation of a wheeze or asthma (Nguyen et al., 2013). Several other studies were also reviewed and revealed that children who received APAP in the first year of life were associated with a significant increase in risk for wheezing later in life. Patients that received APAP regularly for fever had higher risk for more severe, concurrent asthma symptoms (Nguyen et al., 2013). In another study, a clear dose-response relationship was identified between daily use of APAP and increased asthma morbidity (Henderson and Shaheen, 2013). It is unclear if this represents an effect of APAP itself or if the factors precipitating the use of APAP (e.g., upper respiratory tract infections) presage the development of airway disease. Other adverse reactions associated with APAP include Stevens-Johnson Syndrome, and toxic epidermal necrolysis (Kim et al., 2014).

While there have been many proposed mechanisms, the exact mechanism of action of APAP remains unclear. The efficacy of APAP is dose-dependent, which is affected by the PK in the different age groups. This was demonstrated in a study which noted that rescue morphine was used more frequently in patients with lower APAP concentrations (Anderson et al., 1996). Furthermore, it was noted in the same study that children with APAP concentrations about 10 mg/L had a better analgesic response than those with an APAP concentration below 10 mg/L (Anderson et al., 1996).

Differences in APAP efficacy among pediatric and adult populations were also observed. In one study of children ages 10 months to 4 years, it was found that APAP lowered the fevers of some children with febrile seizure and a temperature above 38.5^o^C more effectively than it lowered the fevers of other children (Van Esch et al., 1995). Analysis of the use of APAP to treat children and adolescent patients (ages 4–18) with migraines determined that headaches were reduced by a mean of 1.5 points on a pain scale with the use of APAP after migraine occurrence (Damen et al., 2005). A separate investigation on adults with migraines, APAP was used in combination with caffeine to treat migraine headaches. That study indicated that migraine headache pain was relieved by 1 point on a pain scale (Pini et al., 2012). Another study in adults aged 19–64 with migraine headaches found that APAP reduced pain by 0.6 points on a pain scale (Pini et al., 2008). While pain scales are very subjective measures of efficacy, they indicate that there might be some difference in the efficacy of APAP between children and adults.

### APAP PHARMACOKINETICS IN CHILDREN

As has been observed with many other drugs, the variability in APAP PK varies with age from birth to adulthood. In a study performed on 75 subjects (3 neonates, 25 infants, 25 children, and 22 adolescents), a PK model was developed from data following intravenous APAP administration (Zuppa et al., 2011). The clearance (L/hr) of APAP increased from 2.02 L/hr at 1 month of age to 4.09 L/hr at 1 year of age to 14.27 L/hr at 16 years of age (based on our own analyses on the published mean values with a Spearman correlation, there is a significant correlation between age and APAP clearance, *p* < 0.0001), while the central volume of distribution remained constant at 0.23 L/kg for all ages. Also, the AUC values were 60 to 90% higher than those measured in children and adolescents in the study (Zuppa et al., 2011). Although it is unclear whether this is statistically significant because of the small number of neonates in the study (*n* ≤ 2), the data indicate a possibly large variability in the elimination of APAP in the pediatric population, especially between neonates and older children (Zuppa et al., 2011).

**FIGURE 2:**
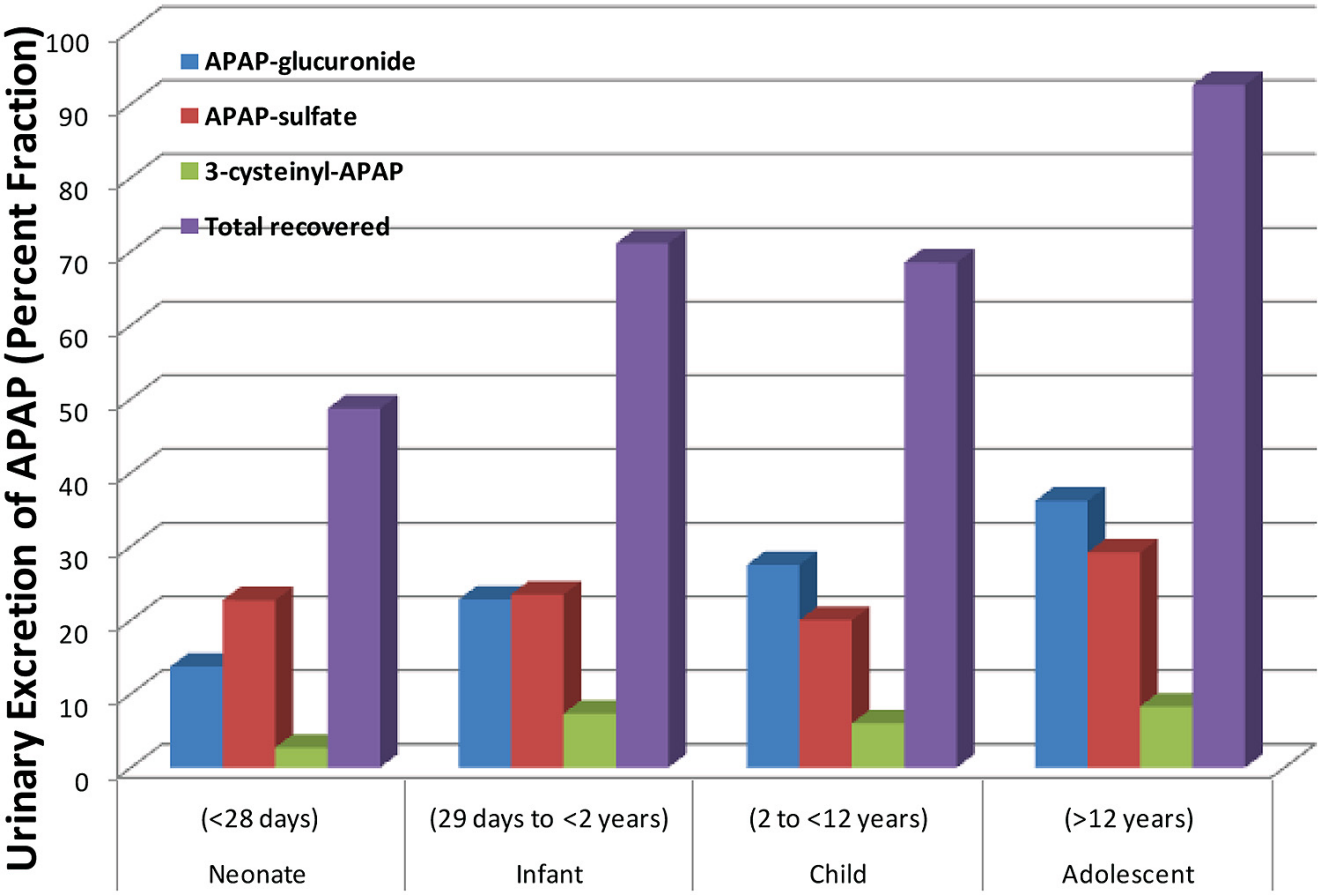
**Differing amounts of the metabolites of acetaminophen that are excreted into the urine across different age groups.** [Data were adapted from Zuppa et al. (2011) with permission]. Shown here is the steady state urinary data presented as percent fractional excretion (y-axis).

APAP metabolism and excretion processes are very complex with both Phase I (oxidation) and Phase II metabolism pathways (glucuronidation, sulfation, and glutathione conjugation) involved (**Figure 1**). In the aforementioned study and illustrated in **Figure 2**, the fraction of APAP-sulfate excreted is fairly similar across age groups, indicating that APAP sulfation is likely constant among all ages (Zuppa et al., 2011). In contrast, the increased fraction of 3-cysteinyl-APAP recovered along with age suggests that there might be an increase in activity of either oxidative or glutathione S-transferase (GST) enzyme isoform activity from neonate to adolescence populations. As expected, the glucuronidation of APAP increased in older children, with a particularly dramatic elevation of over 20-fold observed between the neonate to the infant stage, albeit the statistical significance remains unclear given the small number of neonates in this study. This assessment must be qualified by the recognition that total urinary recovery also varied with age (Zuppa et al., 2011). Other investigators have demonstrated that glucuronide/sulfate ratios are 0.12 in premature neonates ( < 32 weeks), 0.28 in neonates 32–36 weeks post-conception, 0.34 in neonates 0–2 days old, 0.75 in children 3–9 years, and 1.8 in adults (Miller et al., 1976; van der Marel et al., 2003). Taken together, these data indicate an increased role of glucuronidation in the metabolism of APAP during maturation to adolescence. The total fraction of APAP metabolites recovered in the urine increased from 48.7 for neonates to 71.1 for infants, and 92.6 for adolescents (Zuppa et al., 2011).

## MOLECULAR BASIS OF INTER-PATIENT DIFFERENCES

### ACETAMINOPHEN ADME

APAP is absorbed almost completely in the duodenum (McGill and Jaeschke, 2013). It is eliminated in the liver, where the drug is predominantly conjugated to glucuronic acid or sulfate (**Figure 1**). After glucuronidation, the metabolites are excreted in the urine. Glucuronidation is carried out by several UDP-glucuronosyl transferases (UGTs) with the enzymes UGT1A1 and UGT1A6 identified as the most important (Miller et al., 1976). APAP is also largely metabolized by sulfation, which is catalyzed by sulfotransferase (SULT) enzymes (Ward et al., 2001). While sulfation is relatively less understood than glucuronidation, it is known that SULT1A1 is primarily responsible for the sulfation of APAP (Rowden et al., 2005). After therapeutic doses, a small portion of a dose is converted into a reactive metabolite, NAPQI , by the cytochrome P450 (CYP) enzymes, primarily CYP2E1, CYP1A2, and CYP3A4, but also several others (Rowden et al., 2005). In normal dosing, the glutathione-*S*-transferase (GST) enzymes convert NAPQI into acetylamino-2-hydroxyphenyl-glutathione (Zuppa et al., 2011). It is when the GST enzymes are overwhelmed and liver glutathione is depleted that liver toxicity due to APAP occurs (McGill and Jaeschke, 2013). The CYP activities, particularly 1A2, 2E1, and 3A4, play a critical role in APAP-induced liver injury, because they produce the toxic metabolite (**Figure 1**). This has been shown in many studies (Raucy et al., 1989; Lee et al., 1996; Cheung et al., 2005; Gonzalez, 2005). One particular study was performed on three different types of mice: wild-type, *cyp2e1*-null, and *CYP2E1*-humanized mice. When the mice were given 400 mg/kg of APAP, moderate liver necrosis was detected in the wild-type mice. However, it was not detected in the *cyp2e1*-null mice. The same dose given to the *CYP2E1*-humanized mice resulted in severe liver necrosis (Cheung et al., 2005). In another study investigating the role of CYP1A2 in hepatotoxicity, it was found that at doses over 600 mg/kg, *cyp2e1*-null mice experienced hepatotoxicity. This indicates that CYP1A2 and other CYP enzymes are also partially responsible for conversion of APAP to NAPQI (Lee et al., 1996). However, CYP2E1 had a lower K_m_ than CYP1A2 (Snawder et al., 1994; Hazai et al., 2002). In a different study, it was found that inhibition of both CYP2E1 and CYP2A6 significantly decreased the formation of NAPQI (Gonzalez, 2005), suggesting multiple CYPs are involved in the development of APAP hepatotoxicity. Inhibition of CYP1A2 and CYP3A4 did not reduce NAPQI formation (Gonzalez, 2005). These studies broadly suggest that there are several CYPs involved in APAP induced hepatotoxicity (Raucy et al., 1989; Lee et al., 1996; Cheung et al., 2005; Gonzalez, 2005).

### AGE-DEPENDENT CHANGES IN THE APAP METABOLISM PATHWAYS

Among pediatric populations, one important factor influencing inter-patient difference in response to APAP is the ontogeny of various metabolic enzymes. The expression of cytochrome P450 enzymes changes during the development from child to adult. While several P450 enzymes play a role in metabolizing APAP, one particularly relevant enzyme in humans regarding APAP-induced hepatotoxicity is CYP2E1 (Johnsrud et al., 2003). In a developmental protein expression pattern study, human hepatic CYP2E1 expression patterns were determined by Western blot analysis of 238 different human liver samples (Vieira et al., 1996). In these 238 livers, it was found that neonatal CYP2E1 levels were lower than infants aged 31–90 days. The infant CYP2E1 levels were lower than levels found in older children and adults. CYP2E1 protein levels in third trimester fetuses were only 10% of the levels from samples of infants older than 90 days. There was a large variation (80-fold between neonatal samples while only a 4-fold difference was seen within other age groups (Vieira et al., 1996). In another study, postnatal data indicated that infants younger than 90 days old had decreased clearance of CYP2E1 substrates in comparison to older children and adults (Cheung et al., 2005). This would lead to a decreased formation of the toxic metabolite of APAP, which potentially allows younger infants to have a decreased risk of hepatotoxicity. Also, it was found that increased postnatal age and ethnicity, but not gender, were significant factors that affected CYP2E1 developmental expression (McGill and Jaeschke, 2013). Differences in developmental expression were also observed between postnatal infants younger than 90 days and older infants (91 days-18 years of age) and adults (over 18 years of age; Vieira et al., 1996). Other studies in addition to those stated here have also consistently shown that CYP2E1 levels are undetectable in fetal liver, but rise after birth and with increasing age (Court et al., 2001; Lejus et al., 2002). These enzyme expression levels undoubtedly affect APAP metabolism in different age groups. In addition to age, ethnicity may also affect CYP2E1 gene expression. For example, higher CYP2E1 protein content was found in Northern European-Americans and Hispanic-Americans than in African-Americans (Johnsrud et al., 2003). All of these differences could be linked to variability in APAP metabolism.

Differences in APAP glucuronidation by UGT enzymes between individuals can also change the level of susceptibility to APAP toxicity in humans (Strassburg et al., 2002). The hepatic UGT expression level changes from birth to adult age. *UGT1A1* and *1A6* develop differently in the pediatric liver. In a study on UGT expression at different ages, it was found that *UGT1A* and *UGT2B* gene expression was absent in fetal liver at 20 weeks gestation, while *UGT1A1* and *UGT1A6* were present in all samples of pediatric and adult livers, which demonstrates that *de novo* expression of *UGT* genes does not occur after 6 months (Strassburg et al., 2002). *UGT1A9 and UGT2B4* were identified at significantly lower expression in pediatric livers, and regulation of *UGT1A9* expression continues beyond 2 years of age and may continue to affect drug metabolism (Strassburg et al., 2002). In the same study, it was determined that age dependent changes did not occur after 6 months of age between *UGT1A1* and *UGT1A6* expression at either the transcription or the protein level. *UGT* genes are expressed after the fetal/early infant period and the differential upregulation of *UGT* gene expression occurs after 6 months until 24 months of age (Strassburg et al., 2002).

In contrast, studies indicate that human hepatic SULT1A1 is expressed in comparable levels in both fetal and postnatal human livers (Duanmu et al., 2006). The increasing UGT expression in the face of stable SULT expression may be particularly consistent with the aforementioned increasing glucuronide/sulfate ratios from premature neonates to adults (McGill and Jaeschke, 2013).

### POLYMORPHISMS AFFECTING APAP METABOLISM

Besides the ontogenic effect, genetic polymorphisms are potentially an important factor leading to differential toxic responses in APAP metabolism in humans. A *CYP2E1* promoter *RsaI* restriction fragment length polymorphism (RFLP; rs2031920) was associated with half-life of APAP in human subjects in a small cohort study (Ueshima et al., 1996). In the coding region, however, *CYP2E1* polymorphisms are rare, and most of them do not seem to have any direct effect on the enzyme activity (Hu et al., 1997). Three non-synonymous polymorphisms that affect coding sequences have been recently defined. One of these polymorphisms, R76H or CYP2E1*2 (rs72559710), demonstrates decreased catalytic activity. This polymorphism has been identified at a low frequency in Chinese populations, but is not observed in any other ethnic groups (Hu et al., 1997). *In vitro* study suggested that CYP2E1*2 decreases protein expression and catalytic activity of the enzyme (Hu et al., 1997). The CYP2E1*1D (a repeat variant) polymorphism leads to increased CYP metabolic activity and likely explains the differences in ethnicity in CYP2E1 developmental expression (Cheung et al., 2005). Recent expression quantitative traits loci (eQTL) mapping in human liver tissue has suggested that a single nucleotide polymorphism (SNP) rs4512750 located at the *CYP2E1* 3′flanking region is strongly (*p* < 10^-10^) associated with *CYP2E1* mRNA expression in the liver (Schadt et al., 2008). Our *in silico* analysis further found that this polymorphism is in strong linkage disequilibrium (LD) with two polymorphisms (rs2480256 and rs2480257) located at the *CYP2E1* 3′-UTR (untranslated region) which potentially alter microRNA targeting (Wei et al., 2012). However, no study was conducted thus far in testing the association between these polymorphisms and APAP metabolism or toxicity.

Regarding CYP1A2, numerous genetic polymorphisms have been identified, but none of them are currently tested for their relationship with APAP metabolism. CYP3A4 is another major P450 enzymes involved in APAP metabolism. Although several polymorphisms have been observed in the gene region that involved in metabolism of many drugs, no indisputable evidence has been obtained thus far to demonstrate a linkage between these polymorphisms and CYP3A4 activity. On the other hand, CYP3A4 was more inducible with a few transcription factors involved such as pregnane X receptor (PXR), hepatocyte nuclear factor 3-alpha (FoxA2) and peroxisome proliferator-activated receptor-α (PPARA; Lamba et al., 2008; Klein et al., 2012). Previous studies suggested that polymorphisms in these factors may indirectly affect the variability in *CYP3A4* transcription, e.g., *PPARA* rs4253728 (Klein et al., 2012), *PXR* -6944CC (rs2472677), -6513CC (rs6438546), and -4356TT (rs13059232) genotypes (Lamba et al., 2008), as well as *FoxA2* rs1212275 and -415(CGG)_n_ polymorphisms (Lamba et al., 2010). Unfortunately, the relationship between these polymorphisms and APAP metabolism remains unexplored. Since higher inducibility of CYP3A4 increases production of toxic metabolite and may increase the likelihood of APAP-induced hepatotoxicity, the importance of these polymorphisms in APAP pharmacogenetics should be investigated. Additionally, *CYP3A7* is detected at a very high level in human embryonic, fetal, and newborn livers, but is much lower in adult livers (De Wildt et al., 1999). In contrast with these genes, *CYP3A5* has similar activity as *CYP3A4* and alleles clearly influencing CYP3A5 activity have been identified previously, e.g., CYP3A5*3 (rs776746) and CYP3A5*7 (rs41303343; Hustert et al., 2001; Kuehl et al., 2001). In a recent study, it was demonstrated that individuals with the A allele (CYP3A5*1) of the *CYP3A5* rs776746 polymorphism have enhanced formation of NAPQI from APAP compared to individuals with the G allele (CYP3A5*3), who lacked CYP3A5 activity (Court et al., 2014).

Significant work has sought to characterize the effect of polymorphisms in *UGT* genes on APAP. For example, a potential association between SNPs rs10929303, rs1042640, and rs8330 located in the *UGT1A*-3′UTR region and APAP glucuronidation variability was observed in human liver microsomes (Court et al., 2013). These SNPs have greater potential to change disposition of APAP, which is glucuronidated by multiple *UGT1A* isoforms, as the 3′-UTR is shared by all *UGT1A* genes (Court et al., 2013). Of the three *UGT1A*-3′UTR SNPs stated above examined in this study, rs8330 was the most significant SNP related to APAP glucuronidation phenotype at all three concentrations tested in human liver bank samples (0.1, 2, and 40 mM). The rs8330 SNP also demonstrated decreased risk of hepatotoxicity due to APAP glucuronidation. The rs8330 minor allele frequency was most prevalent in African (0.39) and Yoruba (0.50) and least prevalent in white America (0.16) and Asian (0.13; Court et al., 2013). Polymorphisms in *UGT1A6* were also linked to APAP metabolism variability. It was determined that three *UGT1A6* coding region SNPs [S7A (rs6759892), T181A (rs2070959), and R184S (rs1105879)] had a quantifiable effect on glucuronidation (Krishnaswamy et al., 2005a,b). Compared to the UGT1A6*1 (haplotype of multiple alleles), the UGT1A6*2 (haplotype of multiple alleles) allozyme showed two-fold higher intrinsic clearance values. These SNPs are thought to account for 15 to 20% of the 13-fold variability of UGT glucuronidation (Krishnaswamy et al., 2005a,b). The importance of these alleles in APAP pharmacogenetics needs further validation. It should be noted that *UGT1A* genes has a unique genomic organization where there are a tandem array of exons 1 followed by common exons 2–5. Linkage disequilibrium across the entire *UGT1A* locus is generally high (Liu et al., 2005; Maitland et al., 2006). Given the multiple UGTs involved in APAP metabolism, pharmacogenetic research should focus on a locus-wide rather than individual gene (exon 1) level. Our group has recently performed a comprehensive analysis of *UGT* expression quantitative trait loci (eQTLs) in a large set of liver samples, with a number of significant eQTLs identified (Liu et al., 2014). The relationship between these eQTLs and APAP metabolism or toxicity should be considered in future research.

It is also noteworthy that there are several *UGT1A* splicing isoforms (UGT1A_i2) that may repress APAP glucuronidation (Court et al., 2013). Co-expression of the UGT1A6_i2 inhibits glucuronidation of APAP by 90%. In the same study, UGT1A1_i2 inhibited UGT1A1_i1 regulated glucuronidation by nearly 30% (Court et al., 2013). Either of these isoform variants expressed in an individual would be expected to lead to decreased APAP glucuronidation and increased incidence of APAP-induced liver toxicity. However, whether the expression patterns of these isoforms vary among pediatric populations and consequently contribute to inter-patient variability in APAP metabolism or toxicity remains unknown and requires further research.

Polymorphisms in *SULT* and *GST* genes are not as well established, and therefore the existence and effect of such polymorphisms on APAP metabolism are not well known. However, there have been a few studies regarding the effect of such polymorphisms, e.g., SULT1A1*2 (rs9282861) did demonstrate a decreased enzymatic activity compared to *1 (reference haplotype) and *3 (rs1801030), which may indicate a potential for increased risk of hepatotoxicity (Nagar et al., 2006). Copy number variants in *SULT1A1* with functional significance have also recently been reported (Hebbring et al., 2007), but their relationship with APAP metabolism remains unclear. Regarding *GSTP1*, two polymorphisms (rs1695 and rs1138272) have been described. Both are in the substrate binding site and result in amino acid substitutions (Ile105Val and Ala114Val, respectively; Dragovic et al., 2014). These polymorphisms have demonstrated small effects on several substrates’ GSTP1 kinetics, and may not be of any pharmacological or physiological significance (Dragovic et al., 2014). However, a recent study demonstrated that *GSTP1* polymorphism significantly modified risk of wheeze in children with age 5 years who had prenatal exposure to APAP (Perzanowski et al., 2010), while an another study of APAP use versus childhood asthma incidence, maternal *GSTT1* and *GSTM1* but not *GSTP1* genotypes were found to modify the risk of asthma incidence (Shaheen et al., 2010).

It is worth mentioning that the majority of these polymorphisms have significant ethnic difference in their allele frequency according to the HapMap data. Previous studies have demonstrated significant difference in APAP metabolism and hepatotoxicity between ethnicities, e.g., metabolic activation of APAP is significantly lower in Africans than Caucasians (Critchley et al., 1986), while the rate of APAP-induced hepatotoxicity was observed to be low in a large, multi-ethnic Asian population, as compared with reported data in western countries (Marzilawati et al., 2012). Another study also showed that Chinese subjects may possess more rapid APAP absorption, as well as different profile of metabolism via different pathways compared to Caucasians (Critchley et al., 2005). Whether these polymorphisms affect APAP metabolism or toxicity and further account for the ethnic difference has yet to be tested and remains an open question for further investigation.

## CONCLUSION

Significant inter-patient differences in both APAP PK and toxicity profiles have been observed between adults and children as well as between pediatric populations. This variability is attributed to both the ontogenic expression of the APAP metabolism pathway and genetic variations and may be the reason behind variability in toxicities in different age groups. However, a comprehensive evaluation has not been achieved for the ontogeny of all involved genes. The interaction between genetic variants and age-related gene expression has not been determined, which largely limits the pharmacogenomic research for APAP-related clinical outcomes. As a consequence, the development of reliable biomarkers that will aid in the clinical management of APAP toxicity across all ages may be more pressing for identifying pediatric hepatotoxicity, although such indicators have yet to be identified.

## FUTURE RESEARCH

Further research is required in order to identify causes for disparity in APAP metabolism and the resultant induced toxicities amongst pediatric populations and adults. To this end, given the complexity of pediatric APAP pharmacology, a systems based approach is particularly necessary to combine comprehensive investigation of gene ontogeny, pathway- or genome-wide genotyping as well as uncovering gene-age based interactions. As more “omics”-based tools are currently available, it is important to integrate data in multiple levels (e.g., metabolomic studies of APAP would give more detailed information about multiple APAP metabolites and how they change from birth to adulthood). Moreover, integrated genomic (genotype) and transcriptomic/proteomic (expression) data such as eQTL analysis could lead to identifying age-dependent genetic alleles determining mRNA and protein expression differences during human development. For example, high-throughput sequencing represents an efficient strategy to identify rare genetic variants that may confer a larger effect on pharmacogenomic phenotypes. Although the functionality of rare variants may not be easily determined, it has been suggested that the entire spectrum of variants including both common and rare one should be included in routine pharmacogenomic research (Drögemöller et al., 2014). In addition to human studies, cell line-based *in vitro* models and animal models could be also useful for further characterizing the mechanism underlying APAP-induced hepatoxicity. For instance, it has been demonstrated that 3D organotypic culture of the human hepatoma HepRG cells is capable of maintaining hepatic function and is more sensitive to APAP-induced toxicity as compared to the conventional 2D cell culture (Gunness et al., 2013). Sequencing study in two independent cohorts of mouse populations also linked CD44 expression to increased APAP-induced hepatotoxicity (Harrill et al., 2009). Furthermore, the pig has been reviewed to be a promising model as the drug metabolism and toxicities in pigs are similar to those of humans (Puccinelli et al., 2011). The great power of these approaches and models in pharmacogenomic research has been increasingly demonstrated recently, and thus offer great hope for increasing our knowledge regarding ontogenic APAP metabolism and toxicokinetics.

## Conflict of Interest Statement

The authors declare that the research was conducted in the absence of any commercial or financial relationships that could be construed as a potential conflict of interest.
